# Translating in-person care to telehealth: a secondary analysis of GP consultations on musculoskeletal conditions

**DOI:** 10.3399/BJGPO.2024.0013

**Published:** 2025-02-12

**Authors:** Yifu Li, Simon Chan, Lawrence Lu, Tim M Jackson, Hania Rahimi-Ardabili, Annie YS Lau

**Affiliations:** 1 Centre for Health Informatics, Australian Institute of Health Innovation, Macquarie University, Sydney, Australia

**Keywords:** musculoskeletal conditions, telemedicine, telehealth, virtual care, remote consultation, general practice

## Abstract

**Background:**

The COVID-19 pandemic led to a rapid transition to telehealth, particularly in general practice where continuous care for chronic conditions, such as musculoskeletal (MSK), is provided.

**Aim:**

To determine the appropriateness of telehealth for MSK conditions by identifying whether in-person tasks can be supported remotely via telehealth.

**Design & setting:**

This study is a secondary analysis of the Harnessing Resources from the Internet (HaRI) dataset. This dataset comprises of 281 videos of recorded GP consultations. The dataset includes 10 GPs, across eight separate clinics, and was collected during 2017 in the UK.

**Method:**

Content analysis was conducted to identify the clinical tasks, physical examinations, and physical artefacts used during the consultations. A scoring method applying two key metrics was developed to assess the translatability of clinical tasks to telehealth.

**Results:**

Across the 31 MSK consultations analysed, 12 clinical tasks, five physical examinations, and 12 physical artefacts were observed. Of clinical tasks, 17% (*n* = 2/12) were deemed to be ‘easily translatable over telehealth’ and 50% (*n* = 6/12) were deemed ‘relatively easy to be translated over telehealth’. Only 17% (*n* = 2/12) of tasks were rated ‘moderately translatable over telehealth’, and 17% (*n* = 2/12) were deemed ‘potentially translatable over telehealth’. No clinical tasks in this study were categorised as untranslatable to telehealth. The average telehealth translatability score was 7.1/10.

**Conclusion:**

Most clinical tasks observed during in-person GP consultations with patients with MSK conditions are translatable to telehealth. Further research is necessary to investigate the long-term efficacy and safety of telehealth utilisation for MSK conditions in primary care.

## How this fits in

Since the COVID-19 pandemic, there has been a growing body of research into the effectiveness of telehealth among health practitioners. Current literature suggests there is effective management of several MSK conditions by allied health practitioners through telerehabilitation. However, few studies within a primary care setting have investigated the extent to which tasks performed by GPs during in-person MSK consultations are translatable to telehealth. This study aimed to determine the translatability of such clinical tasks observed in this patient cohort.

## Introduction

Musculoskeletal (MSK) conditions currently have the fifth highest impact on life expectancy when dealing with the burden of a disability.^1^ Furthermore, there has been a 11.3% increase in years lived with disabilities per 100 000 people with MSK disorders from 1990–2010 globally.^2^ Such conditions have traditionally been assessed and managed in face-to-face consultations with GPs(^3^). However, there has been a significant rise in the use of telehealth to support GP consultations.^4^ For example, telehealth in Australia only accounted for less than 10% of the total number of GP consultations in the month of March 2020, but the COVID-19 pandemic accelerated the use of virtual communications to be equivalent to the total number of traditional face-to-face consultations by the end of 2020.^5,6^

Previous studies have shown promising results in managing various cardiac or neurological conditions through telehealth.^7,8^ To our knowledge, few studies within a primary care setting have investigated the extent to which tasks observed during in-person GP consultations are translatable to telehealth.^9^ Translatability in this study is defined as the capacity of an action to be conducted in another format (that is, whether a task performed in-person can be transformed to a remote action over telehealth in the form of video or telephone). Chronic MSK conditions require continual management owing to pain, restrictions in activities of daily living, high rates of depression, and generally a lower quality of life.^10^ Thus, there is an important need to identify limits or issues associated with the transition to telehealth. This study aims to identify the clinical tasks, physical examinations, and physical artefacts utilised during in-person GP consultations with patients with MSK conditions through detailed content analysis. We will also analyse whether these aspects are translatable to telehealth and highlight potential knowledge gaps in virtual care suitability.

## Method

### Study design

This study comprises a secondary analysis of GP consultation video recordings and written transcripts. The transcripts were screened for MSK-related consultations, and a translatability score was given to each artefact and examination. The videos are derived from the NHS-ethical approved project: *Harnessing resources from the internet to maximize outcomes for GP consultations (HaRI): a mixed qualitative methods studyP*.^11^ The HaRI study comprises 281 primary care consultations taken from 10 GPs working at eight separate clinics during 2017, at various locations across the UK. See the Ethical approval section for more details.

### Data collection

The HaRI dataset video recordings and transcripts were passed through a list of inclusion and exclusion criteria to restrict the analysis to consultations pertaining to MSK presentations (see Table 1). The inclusion criteria focused on MSK conditions as the primary presentation and with a discussion of lifestyle advice or behavioural modifications. Two researchers (YL, LL) independently read and watched each GP consultation transcript and video. Seventy-five relevant consultations discussing MSK conditions were identified but 29 were removed owing to consent or de-identification issues. A further 15 consultations were removed owing to missing video or written transcripts, leaving 31 eligible consultations for final analysis (see flowchart in Supplementary Figure S1). Note that the dataset does not provide a diagnosis or information on patient outcomes.

**Table 1.** Consultation eligibility criteria

Inclusion criteria	Exclusion criteria
Consultations that involve MSK as part of the patient’s main presentation or past medical history Consultations of patients with MSK conditions that contain any discussion of lifestyle advice, self-management support, or behavioural modifications as part of their treatment or prevention plan MSK consultations across all ages Consultations for which the participants in the video provided consent for researchers to access video recordings and transcripts of their data to be collected in this study	Consultations where MSK was not the main point of discussion between the patient and the GP Consultations where MSK issue(s) did not concern the patient during their visit to the GP Consultations of patients with MSK conditions that does not contain any discussion of lifestyle advice, self-management support, or behavioural modifications as part of their treatment or prevention plan Consultations where the participants in the video did not provide consent for researchers to access video recordings and transcripts of their data to be collected in this study Consultations where a written transcript has been provided but not the video transcript, or vice versa Consultations where the video recording could not be viewed as it was not de-identified, is corrupted, or unavailable owing to other reasons

MSK = musculoskeletal

### Data analysis

#### Descriptive analysis

Descriptive content analysis was applied to eligible video and written transcripts. This included information on the demographics and general characteristics of each consultation. Extracted patient and consultation characteristics were represented using descriptive statistics such as measures of frequency and central tendency.

#### Video and transcript analysis

Two researchers (YL, LL) independently identified the physical examinations and artefacts used by the GP during a MSK consultation. Supplementary Table S1 includes screenshots of examples of physical examinations done during consultations. In all instances where clinical tasks, physical examinations, or physical artefacts were identified, transcript excerpts were noted and allocated to categories inductively formulated during the data-extraction process within an Excel spreadsheet. These categories included the following: (1) physical examinations (for example, upper limb examination); (2) physical artefacts (for example, tendon hammer); and (3) tasks performed during in-person consultations (for example, prescribing medication). Further, two researchers (YL, LL) also re-analysed all 31 videos and extracted the following information: length of entire consultation; number of physical examinations per consultation; and length of time for physical examination(s).

#### Data verification

Two researchers (YL, SC) analysed all videos and transcripts to verify extracted information from the initial data analysis. All information regarding tasks, physical examinations, and physical artefacts were re-extrapolated, verified, and corrected where necessary. Additionally, physical artefacts were further subcategorised into the three groups. This included the following: (1) physical artefacts that are readily found in a patient’s home (for example, computer); (2) physical artefacts that are easily acquired through purchase or provision (for example, sphygmomanometer); and (3) physical artefacts that are not easily acquired by patients in the community (for example, tendon hammer).

#### Translatability to telehealth analysis

A scoring system was utilised to rate the translatability of in-person GP tasks to telehealth. This was partly adopted from a study by Croymans and colleagues,^12^ who developed a rating scale to rank appropriateness of certain conditions to telehealth and was later modified by our team to apply on this dataset to assess other GP conditions such as cardiovascular disease,^13^ respiratory conditions,^14^ and chronic conditions.^15^

**Figure 1.**
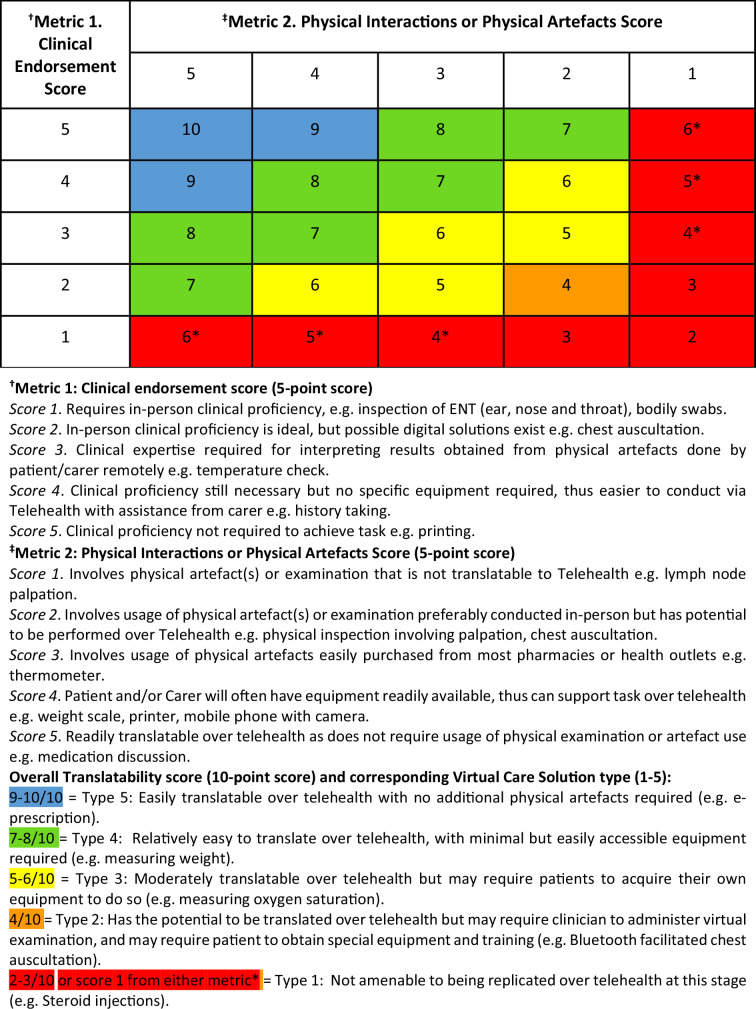
Translatability to telehealth scoring system and type

One researcher (YL) scored tasks according to the translation metrics (see Figure 1), this was then reviewed by the rest of the team. Following this method, tasks were initially assessed based on two 5-point scoring systems to determine if (a) they needed ‘clinical endorsement’ or (b) the extent to which a task could utilise ‘physical artefacts’ remotely. The rationale for the scoring of each task is provided below Figure 1. Lastly, the scores of the two 5-point systems were combined to give an overall score out of 10. This overall score conveys the translatability of a task to telehealth and indicates one of the five potential types of virtual care solution.

## Results

### Demographics and consultation characteristics

Among 31 MSK consultations, 61.3% (*n* = 19) of patients were female, and the majority of patients were aged 66–75 years at 32.3% (*n* = 10). Additionally, 67.7% of the consultations discussed a singular health condition (*n* = 21). Some examples of the MSK conditions discussed include lateral epicondylitis, osteoarthritis, and tendonitis (See Table 2).

**Table 2.** Characteristics of consultations and patients with a MSK presentation (*n* = 31)

Variables	MSK consultations (*n* = 31)
**Sex % (** ***n*** **)**	
Male Female	38.7% (12) 61.3% (19)
**Age in years % (** ***n*** **)**	
0−18 19−35 36−45 46−55 56−65 66−75 ≥76	6.5% (2) 6.5% (2) 6.5% (2) 19.4% (6) 19.4% (6) 32.3% (10) 9.7% (3)
**Presence of a companion % (** ***n*** **)**	
Yes No	29.0% (9) 71.0% (22)
**Number of health conditions discussed during consultation % (** ***n*** **)**	
1 2 3	67.7% (21) 25.8% (8) 6.5% (2)
Types of MSK conditions discussed	Disc herniation, multilevel degenerative disc disease, gout, carpal tunnel syndrome, lateral epicondylitis, non-specific lower back pain, coccydynia, reactive arthritis, rheumatoid arthritis, osteoarthritis, plantar fasciitis, sciatica, osteopenia, soft tissue injury or pain, medial epicondylitis, bursitis, tendonitis, fracture, De Quervain’s tenosynovitis.
Other health conditions discussed	GORD, urinary frequency, dementia, breast cancer, hypertension.
Objective measures used to assess MSK conditions	History-taking, past medical history, physical exams, blood pressure, weight, blood tests, BMD scan, X-rays, MRI, CT.
Subjective conditions used to assess MSK conditions	Patient mood, exercise tolerance, ADLs, pain and current pain control, sleep quality.

ADLs = activities of daily living. BMD = bone mineral density. CT = computed tomography. GORD = gastro-oesophageal reflux disease. MRI = magnetic resonance imaging. MSK = musculoskeletal

### Physical examinations in GP consultations

Five types of physical examinations were conducted and 100% of consultations (*n* = 31) were observed to involve a general visual inspection of the patient. Following this, palpation and movement examinations were observed in 83.9% (*n* = 26) of consultations. Blood pressure measurement then followed at 16.1% (*n* = 5), weight measurement at 6.5% (*n* = 2), and neurological examination at 6.5% (*n* = 2).

To provide context on the consultations, frequency and time analysis has been included, which showed the average total length of all the GP consultations was 10 minutes 44 seconds. The average time spent on physical examination(s) during a consultation was 1 minute 7 seconds (Supplementary Table S3 for details). Our findings revealed that the time spent on physical examination(s) only comprised a small proportion of the total consultation time (10.4%).

### Physical artefacts used during in-person consultations

**Figure 2.**
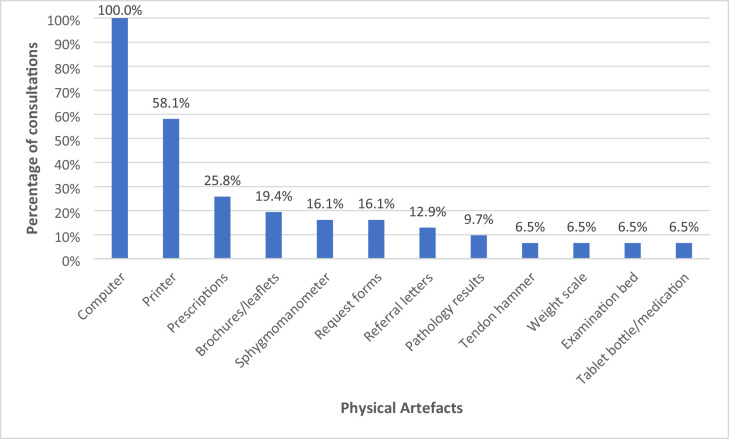
Types and frequency of physical artefacts used during in-person musculoskeletal (MSK) consultations, as a percentage of all GP consultations involving a MSK condition (*n* = 31)

The frequency of physical artefacts observed across in-person MSK consultations is outlined in Figure 2. Overall, 12 distinct physical artefacts were identified across these 31 consultations. A computer was utilised in 100% of all consultations, followed by a printer (58.1%), prescription script (25.8%), medical brochures or leaflets (19.4%), sphygmomanometer (16.1%), request forms (16.1%), referral letters (12.9%), pathology results (9.7%), tendon hammer (6.5%), weight scale (6.5%), examination bed (6.5%), and medications (6.5%).

Of the 12 physical artefacts observed, three were defined as readily found in a patient’s home setting, three were defined as easily acquired through purchase or provision, and six were defined as not easily acquired. These are as follows:

- computer, printer, and weight scale were readily found in a patient’s home setting;
- brochures or leaflets, sphygmomanometer, and medication(s) were easily acquired through purchase or provision;
- tendon hammer, prescriptions, request forms, referral letters, pathology results, and examination bed were not readily found or easily acquired.

### Clinical tasks performed during in-person consultations

**Figure 3.**
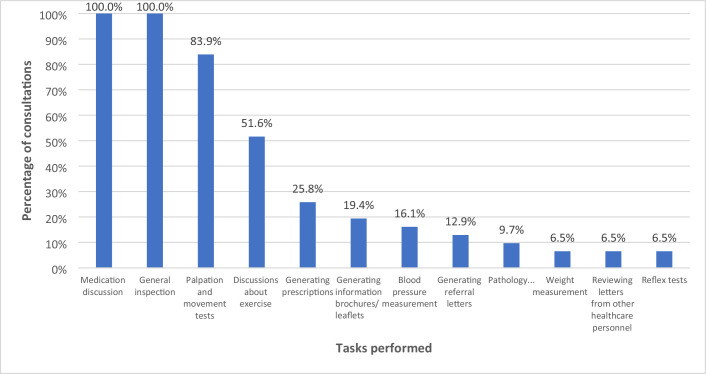
Types and frequency of tasks performed during in-person musculoskeletal (MSK), as a percentage of all GP consultations involving a MSK condition (*n* = 31)

A total of 12 clinical tasks were observed across 31 MSK consultations (See Figure 3). Out of the 12 tasks performed, 100% involved discussion of a prescribed and/or over-the-counter medication (for example, amitriptyline, tramadol, paracetamol) and a general inspection. While 83.9% (*n* = 26) of consultations involved some sort of physical examination (for example, palpation, range of movement, tendon reflexes). Discussions about exercise and lifestyle modifications comprised 51.6% (*n* = 16) of consultations while 25.8% (*n* = 8) involved a new or continued prescription of medication(s).

### Translatability of tasks to telehealth

Table 3 presents scores indicating the degree of translatability to telehealth for all 12 tasks observed across GP consultations on MSK conditions. On average, scores for the clinical endorsement, physical interaction, and combined translatability to telehealth were 3.4/5, 3.7/5, and 7.1/10, respectively. Many of the tasks were non-MSK specific but rather GP-related tasks (for example, generating prescriptions). The telehealth solution categories were assigned to each task were as follows:

- no task was rated as type 1 (not amenable to being translated to telehealth at this stage).
- 17% (*n* = 2/12) of tasks were rated as type 2 (has some potential to be translated over telehealth): palpation and movement tests, reflex tests.
- 17% (*n* = 2/12) of tasks were rated as type 3 (moderately translatable over telehealth): blood pressure measurement, general inspection.
- 50% (*n* = 6/12) of tasks were rated as type 4 (relatively easy to translate over telehealth): weight measurement, generating prescriptions, generating referral letters, pathology test interpretationor discussion, medication discussion, and reviewing letters from other healthcare personnel.
- 17% (*n* = 2/12) of tasks were rated as type 5 (easily translatable over telehealth): generating information brochures or leaflets, and lifestyle discussion.

**Table 3.** Clinical tasks conducted during in-person musculoskeletal (MSK) consultations with the GP (*n* = 31). Clinical endorsement scores, physical examinations or artefacts scores and combined translatability to telehealth scores are assigned to the 12 clinical tasks observed during consultations

Clinical task	Physical artefact(s)	Clinical endorsement score	Physical examination or artefacts score	Translatability to telehealth score	Virtual care solution type	MSK specific?	Implication
**Physical examination**							
Blood pressure	Sphygmomanometer	3	3	6/10	Type 3	No	Moderately translatable to telehealth
Weight measurement	Weight scale	3	4	7/10	Type 4	No	Relatively easy to translate to telehealth
General inspection	Nil	2	4	6/10	Type 3	No	Moderately translatable to telehealth
Palpation and movement tests	Examination bed	2	2	4/10	Type 2	Yes	Has the potential to be translated to telehealth
Reflex tests	Tendon hammer	2	2	4/10	Type 2	Yes	Has the potential to be translated to telehealth
**Management and/or investigation**							
Generating prescriptions	Computer, printer	4	4	8/10	Type 4	No	Relatively easy to translate to telehealth
Generating information brochures or leaflets	Computer, printer	5	4	9/10	Type 5	No	Easy to translate over to telehealth
Generating referral letters	Computer, printer	4	4	8/10	Type 4	No	Relatively easy to translate to telehealth
Pathology test interpretation or discussion	Computer	4	4	8/10	Type 4	No	Relatively easy to translate to telehealth
Reviewing letters from other healthcare personnel	Computer	4	4	8/10	Type 4	No	Relatively easy to translate to telehealth
Medication discussion	Tablet bottle	4	4	8/10	Type 4	No	Relatively easy to translate to telehealth
Lifestyle discussion	Nil	4	5	9/10	Type 5	No	Easy to translate over to telehealth

MSK = musculoskeletal

## Discussion

### Summary

The average translatability to telehealth score was 7.1 out of 10. This indicates that on average MSK consultations in primary care were ‘relatively easy to translate over telehealth’. The majority of physical examinations required easily accessible equipment for the patient or did not require a high degree of in-person clinical expertise and complexity. Our findings revealed that the time spent on physical examination(s) only comprised a small proportion of the total consultation time (10.4%). Furthermore, most clinical tasks observed were non-MSK specific. These findings suggest that telehealth could be an appropriate fit with MSK patient management in primary care.

### Strengths and limitations

The major strength of this study was that it analysed data from real-life GP consultations via video and written transcripts, instead of using self-reported data such as interviews or surveys. It used a unique dataset of multiple GPs across different GP clinics, and an objective analysis of these MSK consultations minimised the potential for recall bias and measurement errors commonly associated with self-reporting methods. This study also involved multiple analysts and coders, therefore reducing the likelihood of measurement errors or subjective biases.

A limitation of the study was that findings were limited to a sample size of 31 consultations, whereby a small sample size may not be an accurate reflection of the scope of clinical tasks and artefacts utilised during MSK consultations in general practice. This also limits the investigation into the dynamic between the GP and the patient, which could be explored in future research. Furthermore, all consultations took place in an English-speaking country, potentially limiting the generalisation of our findings to other contexts outside of the NHS health system. Finally, clinical endorsement and physical artefact scores may vary dependent on the patient’s and GP’s ability and willingness to use telehealth. This could be further explored in a comparison between in-person and virtual consultations.

### Comparison with existing literature

This dataset has previously been examined for telehealth appropriateness with the scoring method for other conditions. The studies include consultations for diabetes (7.2/10),^16^ respiratory (6.7/10),^13^ and chronic conditions (7.0/10).^14^ The secondary analysis by Lane and colleagues^16^ showed similar virtual care solution types to our study for palpation examinations, movement tests, and lifestyle discussions.

In a similar methodology, the Telehealth Appropriateness by Patient Concerns scale developed by Croymans and colleagues^12^ conveyed that joint problems or pain scored 5.3 out of 9, indicating that it was slightly skewed towards ‘appropriate’ for telehealth. Furthermore, a randomised trial by Odole and Ojo,^15^ investigating the effects of a 6-week telephysiotherapy programme for patients with knee osteoarthritis, found that improvements in quality of life were comparable with in-person treatment. Their results reaffirm our findings that certain key aspects of lifestyle discussion and continued management are ‘easy to translate over telehealth’, particularly as physical examinations required easily accessible equipment and comprised only a small proportion of total consultation time. Also, a primary study by Russell *et al* ^17^ concluded that assessments of non-articular lower limb conditions were reliable and viable to traditional in-person examinations. These results could also be boosted by the large-scale uptake of wearable devices in the wake of the COVID-19 pandemic.^18,19^

Studies have found that assessment and diagnosis of shoulder joints were poor, as well as when determining reasons for limited lumbar range of motion and postural analysis for low back pain.^20,21^ Such discrepancies are likely owing to differences in methodology, as these studies examined the validity of investigating undifferentiated MSK diagnoses via telehealth. In comparison, our study comprised of patients who were examined by the GP for ongoing review and management, rather than formulating a principal diagnosis. Initial assessment and diagnosis typically require a higher degree of in-person clinical assessment and can involve more complex or highly specific physical examinations. However, there has been an increase in artificial intelligence (AI) research in the diagnostic process, which could increase the translatability of in-person interactions.^22,23^ This is in addition to the new advances in augmented and virtual reality solutions for the treatment of MSK disorders.^24–26^

### Implications for research and practice

The majority of MSK consultations involved at least one type of physical examination, commonly in the form of an inspection. However, most consultations required little to no use of easily accessible equipment or did not require in-person clinical expertise. Our study also found that the time spent by GPs on performing specific examinations only comprised a very small proportion of the total consultation length. These findings indicate good potential for the translatability to telehealth within the primary care setting, owing to the lesser need to perform complex exams remotely.

Our study has identified several knowledge gaps. Further high quality randomised trials comparing telemedicine with control groups to investigate efficacy across all MSK disorders are needed to maximise the potential of this technology. There is also existing guidance to assist clinicians on performing a virtual MSK examination for a variety of tests.^27^ However, some safety concerns arise as there is a risk that remote assessments may be inaccurate and precipitate care that is ineffective or inefficient. The sensitivity and specificity of these self-performed tests, as well as patient satisfaction, have yet to be documented extensively in the literature.^27^ In addition, a recent scoping review found that the costs of telehealth solutions for MSK disorders are rarely researched.^28^

In conclusion, overall assessment of 12 tasks observed in GP consultations on MSK care indicated that tasks were ‘relatively easy to translate over telehealth’. These tasks did not require a high degree of in-person clinical expertise and can be accomplished with easily accessible equipment, indicating a good potential for telehealth solutions for MSK GP consultations. Future studies may further validate our findings, and examine safety concerns and quality-of-care outcomes in real-world telehealth interventions.

## Data

The dataset on which the conclusions of this article rely is not publicly available.

## References

[1] GBD 2017 DALYs and HALE Collaborators (2018). Global, regional, and national disability-adjusted life-years (DALYs) for 359 diseases and injuries and healthy life expectancy (HALE) for 195 countries and territories, 1990–2017: a systematic analysis for the Global Burden of Disease Study 2017. Lancet.

[2] Vos T, Flaxman AD, Naghavi M (2012). Years lived with disability (YLDs) for 1160 sequelae of 289 diseases and injuries 1990–2010: a systematic analysis for the Global Burden of Disease Study 2010. Lancet.

[3] Australian Institute of Health and Welfare (2019). Chronic musculoskeletal conditions: Back problems.

[4] Haas R, Busija L, Gorelik A (2021). Patterns of care for people presenting to Australian general practice with musculoskeletal complaints based on routinely collected data: protocol for an observational cohort study using the Population Level Analysis and Reporting (POLAR) database. BMJ Open.

[5] Pearce C, McLeod A, Gardner K (2020). The GP Insights Series no 7: primary care and SARS-CoV-2: the first 40 weeks of the pandemic year.

[6] Jonnagaddala J, Godinho MA, Liaw S-T (2021). From telehealth to virtual primary care in Australia? A rapid scoping review. Int J Med Inform.

[7] Sari DM, Wijaya LCG (2021). Cardiac rehabilitation via telerehabilitation in COVID-19 pandemic situation. Egypt Heart J.

[8] Cramer SC, Dodakian L, Le V (2020). A feasibility study of expanded home-based telerehabilitation after stroke. Front Neurol.

[9] Wong B, Ward D, Gemmell K (2020). How is telehealth being utilized in the context of rehabilitation for lower limb musculoskeletal disorders: a scoping review. Phys Ther Rev.

[10] Şahin N, Devrimsel G, Karahan AY, Sargın S (2020). The effects and characteristics of musculoskeletal pain on quality of life in geriatric patients. European Journal of Geriatrics and Gerontology.

[11] Seguin M, Hall L, Atherton H (2018). Protocol paper for the “Harnessing resources from the internet to maximise outcomes from GP consultations (HaRI)” study: a mixed qualitative methods study. BMJ Open.

[12] Croymans D, Hurst I, Han M (2020). Telehealth: the right care, at the right time, via the right medium. https://catalyst.nejm.org/doi/full/10.1056/CAT.20.0564.

[13] Raghuraman S, Ramarao J, Lane J (2023). Analysis of in-person general practice respiratory consultations: assessing translatability to telehealth. BJGP Open.

[14] Ward K, Vagholkar S, Lane J (2023). Are chronic condition management visits translatable to telehealth? Analysis of in-person consultations in primary care. Int J Med Inform.

[15] Odole AC, Ojo OD (2014). Is telephysiotherapy an option for improved quality of life in patients with osteoarthritis of the knee?. Int J Telemed Appl.

[16] Lane J, David K, Ramarao J (2023). Translating primary care to telehealth: analysis of in-person consultations on diabetes and cardiovascular disease. BJGP Open.

[17] Russell TG, Blumke R, Richardson B, Truter P (2010). Telerehabilitation mediated physiotherapy assessment of ankle disorders. Physiother Res Int.

[18] Östlind E, Eek F, Stigmar K (2022). Promoting work ability with a wearable activity tracker in working age individuals with hip and/or knee osteoarthritis: a randomized controlled trial. BMC Musculoskelet Disord.

[19] Saito Y, Ishida T, Kataoka Y (2022). Evaluation of gait characteristics in subjects with locomotive syndrome using wearable gait sensors. BMC Musculoskelet Disord.

[20] Steele L, Lade H, McKenzie S, Russell TG (2012). Assessment and diagnosis of musculoskeletal shoulder disorders over the internet. Int J Telemed Appl.

[21] Truter P, Russell T, Fary R (2014). The validity of physical therapy assessment of low back pain via telerehabilitation in a clinical setting. Telemed J E Health.

[22] Esfandiari H, Troxler P, Hodel S (2022). Introducing a brain-computer interface to facilitate intraoperative medical imaging control — a feasibility study. BMC Musculoskelet Disord.

[23] Olsson S, Akbarian E, Lind A (2021). Automating classification of osteoarthritis according to Kellgren-Lawrence in the knee using deep learning in an unfiltered adult population. BMC Musculoskelet Disord.

[24] Brady N, Lewis J, McCreesh K (2021). Physiotherapist beliefs and perspectives on virtual reality-supported rehabilitation for the assessment and management of musculoskeletal shoulder pain: a focus group study protocol. HRB Open Res.

[25] Rutkowski S, Kiper P, Cacciante L (2020). Use of virtual reality-based training in different fields of rehabilitation: a systematic review and meta-analysis. J Rehabil Med.

[26] Gumaa M, Rehan Youssef A (2019). Is virtual reality effective in orthopedic rehabilitation? A systematic review and meta-analysis. Phys Ther.

[27] Laskowski ER, Johnson SE, Shelerud RA (2020). The telemedicine musculoskeletal examination. Mayo Clin Proc.

[28] Bargeri S, Castellini G, Vitale JA (2024). Effectiveness of telemedicine for musculoskeletal disorders: umbrella review. J Med Internet Res.

